# Potential pitfalls in claiming novel taxa

**DOI:** 10.1002/imt2.70036

**Published:** 2025-04-19

**Authors:** Rashidin Abdugheni, Shuang‐Jiang Liu

**Affiliations:** ^1^ Department of Microbiology, School of Basic Medical Sciences Xinjiang Medical University Urumqi China; ^2^ State Key Laboratory of Microbial Technology Shandong University Qingdao China; ^3^ State Key Laboratory of Microbial Diversity and Innovative Utilization, and Environmental Microbiology Research Center (EMRC), Institute of Microbiology Chinese Academy of Sciences Beijing China

## Abstract

Prokaryotic taxonomy based on short 16S rRNA sequences may lead to an overestimation of microbial diversity. In addition, a lack of sufficient coverage of previously reported taxa may lead to repetition or overestimation of novel taxa. In light of a recent study published in iMeta, we have issued a comment to remind microbial taxonomists of the importance of maintaining rigor and precision when delineating microbial species, urging researchers to avoid imprecise approaches.

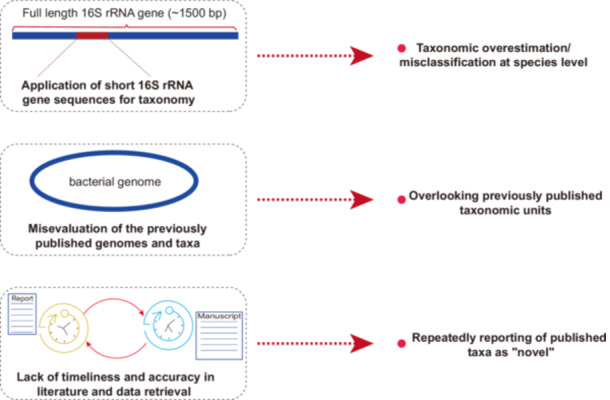

To the Editor,

With the application of Next‐Generation Sequencing (NGS), bacterial isolation and culturing, and the collection of numerous samples from humans, animals, and various environments, many novel taxa and their refined classifications have been proposed. The advent of the NGS and sophisticated bioinformatic tools has enabled a more precise framework for prokaryotic classification and taxonomic revisions using high‐quality genome sequences. Notable examples include proposal of novel taxa of *Segatella copri* [1], *Faecalibacterium* [2], and refined classification of *Nocardiopsis* [3], *Bifidobacterium faecale* [4], *Arachnia propionica* [5], and members of the *Listeriaceae* family [6] by in‐depth phylogenomic analysis and genome‐based taxonomy. However, claiming novel taxa requires appropriate methodologies and a reliable and complete data source, including a comprehensive evaluation of sufficient genetic and phenotypic aspects of the isolated microorganisms. We would like to discuss several potential pitfalls in claiming novel taxa. To do so, we used a research study as an example, which is titled “Isolation of potentially novel species expands the genomic and functional diversity of *Lachnospiraceae*” (iMeta. 2024; 3:e174.), authored by Lin et al. [7].

Firstly, a lack of sufficient coverage of previously reported taxa may lead to repetition or overestimation of novel taxa. For example, the same 54 microbial taxa cultured in 2016 [8] have been repeatedly reported as “novel” in another research in 2019 [9]. This oversight likely arises from researchers' inadequate evaluation and incomplete search of previous literature when assessing their own findings, leading to species redundancy. A similar issue can be found in the research article by Lin et al. [7] who reported the collection of 1,868 high‐quality genomes, including 756 genomes from their isolated strains, of the *Lachnospiraceae* family. Lin et al. claimed that “*We found that 47.88% of the newly cultured genomes in CGR2 were potentially novel species, and 22.22% corresponded to potentially novel genera, using similarities of 98.7% and 94.5% as the species and genus demarcation* [10], *respectively. In addition, the 16S rRNA gene sequences of the potentially novel genera were clustered into 37 genus‐level operational taxonomic units (OTUs) and 64 species‐level OTUs. Notably, the genomes from CGR2 not only covered most genera of the Lachnospiraceae family identified in the human gut microbiota but also added three potentially new genera that had not been isolated previously from the human gut (*Figure 1A *)*”. Here, readers have found that the claim of “*newly cultured genomes in CGR2 were potentially novel species*” was misleading and overestimating, as many of the genomes had been previously published and taxonomically defined before Lin's publication [7] (for details, please refer Table 1 of this communication). As listed in Table 1, readers found that 26 clusters claimed as representing novel species by Lin et al. [7] were previously reported in culture‐based studies, and 25 of these species listed in Table 1 were reported even before genome collection of Lin et al. (which was stated as July 2021). We consider it inappropriate to disregard prior efforts, reports, and scientific literatures.

**Figure 1:**
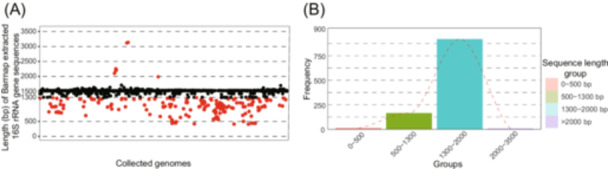
Demonstration of the 16S rRNA gene sequence features of Lachnospiraceae extracted by Lin et al. [7] using the Barrnap. (A) Distribution of the extracted Lachnospiraceae 16S rRNA gene sequences. There are 176 genomes with a sequence length shorter than 1300 bp and 11 genomes with a sequence length even shorter than 500 bp. Notice that six genomes have extremely long 16S rRNA gene sequences (>2000 bp), which are longer than the entire 16S rRNA gene (~1500 bp). (B) Histogram categorizing sequences into four length groups (0–500 bp, 500–1300 bp, 1300–2000 bp, and >2000 bp), with frequencies indicated on the *y*‐axis.

**Table 1:** Repeated claim of “novel species” of *Lachnospiraceae* that had been previously reported.

No.	Cluster IDs claimed representing novel taxa by Lin et al. [7]	Previously reported species names, genome accessions, published dates (month/year), and references
1	1	*Blautia difficilis*, GCA_014297245, 05/2021, [11]
2	10	*Anaerostipes hominis*, GCA_014290215, 05/2021, [11]
3	31	*Mediterraneibacter massiliensis*, GCA_001487105, 05/2021, [11]
4	32	*Coprococcus hominis*, GCA_014287735, 05/2021, [11]
5	35	*Simiaoa sunii*, GCA_014337175, 05/2021, [11]
6	41	*Clostridium minihomine*, GCA_900290215, 03/2018, [12]
7	64	*Blautia celeris*, GCA_014287615, 05/2021, [11]
8	66	*Lachnoclostridium edouardi*, GCA_900240245, 05/2017, [13]
9	78	*Coprococcus phoceensis*, GCA_900104635, 04/2019, [14]
10	188	*Lachnoclostridium phocaeense*, GCA_018223505, 01/2017, [15]
11	214	*Blautia marasmi*, GCA_900258535, 05/2017, [16]
12	239	*Africanella massiliensis*, GCF_900086625, 07/2016, [17]
13	262	*Dorea phocaeensis*, GCA_900240315, 09/2019, [18]
14	268	*Mediterraneibacter hominis*, GCA_014287475, 05/2021, [11]
15	269	*Roseburia rectibacter*, GCA_014287515, 05/2021, [11]
16	270	*Anaerosacchariphilus hominis*, GCA_014290175, 05/2021, [11]
17	270	*Roseburia difficilis*, GCA_014287625, 05/2021, [11]
18	272	*Dorea hominis*, GCA_014287705, 05/2021, [11]
19	274	*Wujia chipingensis*, GCA_014337155, 05/2021, [11]
20	275	*Qiania dongpingensis*, GCA_014337195, 05/2021, [11]
21	276	*Wansuia hejianensis*, GCA_014337215, 05/2021, [11]
22	277	*Jutongia huaianensis*, GCA_014384985, 05/2021, [11]
23	279	*Jingyaoa shaoxingensis*, GCA_014385005, 05/2021, [11]
24	281	*Lachnoclostridium phocaeense*, GCA_900120345, 01/2017, [15]
25	335	*Konateibacter massiliensis*, GCA_900184995, 01/2022, [19]
26	342	*Lachnoclostridium massiliosenegalense*, GCF_900078195, 08/2016, [20]

Secondly, relying exclusively on the 16S rRNA gene sequence for taxonomic assignment is insufficient to reach a reliable taxonomic conclusion. Depending exclusively on one single taxonomic indicator, such as 16S rRNA gene identity, may yield inaccurate taxonomic conclusions or/and overestimated taxonomic units. An in‐depth genome and 16S rRNA gene‐based taxonomic analysis of *Actinobacteria* found the inconsistency of the taxonomic lineages and overestimation of species while referencing merely on the 16S rRNA gene‐based taxonomic conclusions [21]. Another recent study on the taxonomy of *Streptomyces* by evaluating the lineages concluded from the 16S rRNA gene and from whole genomes also revealed disagreement in taxonomic conclusions [22]. Likewise, Lin's taxonomic assignment of clusters based on the partial 16S rRNA sequences extracted from genomes is inaccurate when applied to conclude species‐level taxonomy, and yielded overestimated false results. Lin et al. [7] applied 98.7% and 94.5% threshold values of 16S rRNA gene similarities for species and genus delineation, respectively. As clearly specified by Yarza et al. [23], which was cited by Lin et al. [7], those values would be robust only when the 16S rRNA gene sequences are at least 1300 bp. In the work of Lin et al., there are 176 genomes with 16S rRNA gene sequences are shorter than 1300 bp, and 11 genomes with sequence lengths are even shorter than 500 bp (Figures 1A,B, this communication), which increases the risk of errors and inaccuracies in assigning those genomes into taxonomic units. In previous reports, Johnson et al. demonstrated that targeting short 16S rRNA sequences cannot provide the taxonomic resolution achievable through sequencing the entire 16S rRNA gene (~1500 bp) [10, 24]. Therefore, the assignment of 1868 genomes to 387 species‐level taxonomic units by Lin et al. [7] has overestimated the actual number of species within the family *Lachnospiraceae*.

Additionally, the classification and reporting of the novel taxonomy of microorganisms requires both timeliness and accuracy. Readers also noticed that Lin's manuscript was submitted in November 2023, but the data included in the manuscript was limited to July 2021, which overlooked significant latest updates in related research fields and is not a common practice in scientific activities. Extensively related to the paper of Lin et al. [7], there were 176 species [25] and 90 genera of *Lachnospiraceae* (by December of 2023, https://lpsn.dsmz.de/family/Lachnospiraceae), and Lin et al. stated only 58 genera in their article [7]. Being readers of Lin's publication [7], we alert researchers of this field to refer to the latest progress in microbial taxonomy and be rigorous and cautious, particularly while claiming novel taxa based solely on the 16S rRNA gene or genome sequences. Also, we urge that researchers must collect up‐to‐date data, and the evaluation and analysis of data must be rigorous, accurate, reliable, and timely to avoid misleading or biased results and conclusions. This is especially critical when referencing existing standards and guidelines, such as in the case of taxonomic assignments, proposals, and validation of novel taxa of prokaryotes.

In summary, microbial taxonomy is a rigorous and comprehensive research endeavor. In the past decades, prokaryotic taxonomy has been evolving toward a comprehensive incorporation of polyphasic aspects by the inclusion of conserved‐gene‐based phylogenetics (e.g., 16S rRNA genes) [26], genome‐based phylogenomics (e.g., average nucleotide identity, ANI) [27] and phenotypic features. Polyphasic taxonomy collects sufficient taxonomic evidence to draw robust taxonomic conclusions and to avoid species redundancy and misclassification. Hence, microbiologists should apply robust data and refer to the latest taxonomic requirements and updates to conduct comprehensive taxonomic investigations and by using filtered, high‐quality data to derive accurate, reliable, and sufficiently comprehensive but not redundant taxonomic conclusions.

## AUTHOR CONTRIBUTIONS

**Rashidin Abdugheni**: Methodology; visualization; writing—original draft; investigation; formal analysis; data curation. **Shuang‐Jiang Liu**: Supervision; writing—review and editing.

## CONFLICT OF INTEREST STATEMENT

The authors have worked on and published papers related to *Lachnospiraceae* [11, 25, 28, 29].

## ETHICS STATEMENT

No animals or humans were involved in this study.

## Supporting information

The data in this table is derived from the Supplementary Table S1 of the article by Lin et al. (https://onlinelibrary.wiley.com/doi/full/10.1002/imt2.174).

## Data Availability

Data sharing is not applicable to this article as no new data were created or analyzed in this study. The data included in the analysis and as a reference are stored in Table S1. Supplementary materials (tables, graphical abstract, slides, videos, Chinese translated version, and update materials) may be found in the online DOI or iMeta Science http://www.imeta.science/.
